# Prevalence of Carbapenem-Resistant Enterobacteriaceae in Western Saudi Arabia and Increasing Trends in the Antimicrobial Resistance of Enterobacteriaceae

**DOI:** 10.7759/cureus.35050

**Published:** 2023-02-16

**Authors:** Rbab Taha, Abdulfattah Mowallad, Areej Mufti, Abdulhakeem Althaqafi, Asif A Jiman-Fatani, Dalia El-Hossary, John Ossenkopp, Baraa AlhajHussein, Mai Kaaki, Noha Jawi, Ashraf Hassanien, Asim Alsaedi

**Affiliations:** 1 Transplant Infectious Disease, King Faisal Specialist Hospital and Research Center, Jeddah, SAU; 2 Pathology and Laboratory Medicine, King Saud Bin Abdulaziz University for Health Sciences, Jeddah, SAU; 3 College of Medicine, King Saud Bin Abdulaziz University for Health Sciences, Jeddah, SAU; 4 Infectious Diseases, King Abdullah International Medical Research Center, Jeddah, SAU; 5 Department of Medical Microbiology and Parasitology, Faculty of Medicine, King Abdulaziz University, Jeddah, SAU; 6 Clinical and Molecular Microbiology Laboratory, King Abdulaziz University Hospital, Jeddah, SAU; 7 Medical Microbiology and Immunology Department, Faculty of Medicine, Zagazig University, Zagazig, EGY; 8 Infection Prevention and Control, King Saud Bin Abdulaziz University for Health Sciences, Jeddah, SAU; 9 Anti-infectives, Pfizer, Jeddah, SAU; 10 Infection Prevention and Control Department, King Saud Bin Abdulaziz University for Health Sciences, Jeddah, SAU

**Keywords:** e. coli., k. pneumoniae, blaoxa-48, blaimp, blandm, blavim, carbapenemase

## Abstract

Purpose: The aim of the study is to estimate the prevalence rate of carbapenem-resistant Enterobacteriaceae (CRE) and to determine the types of carbapenemase genes present in patients admitted to King Abdulaziz Medical City (KAMC-J) and King Abdulaziz University Hospital (KAUH), both in Jeddah, Saudi Arabia.

Methods: A total of 180 isolates were analyzed which were included on the basis of retrospective chart review of patients from KAMC-J and KAUH between 1^st^ April 2017 to 30^th^ March 2019. The prevalence of carbapenemase genes* (^bla^IMP, ^bla^VIM, ^bla^KPC, ^bla^NDM-1, and ^bla^OXA-48) *was evaluated by Xpert® Carba-R (Cepheid, Sunnyvale, CA, USA). We assessed the CRE prevalence and described their susceptibility to antimicrobial agents based on antibiogram reports.

Results: *Klebsiella pneumoniae* showed a higher frequency of *^bla^*OXA-48 (79%) than *^bla^*NDM (11.7%) genes (p=0.007). The CRE prevalence in KAUH was 8% in 2017 and increased to 13% in 2018. In KAMC-J, the prevalence was 57% in 2018 and 61% in 2019. *K. pneumoniae* was found to be the most frequently isolated causative organism followed by *Escherichia coli.* The *^bla^*OXA-48 (76.1%) gene was predominant among overall isolates followed by *^bla^*NDM (13.9%); both genes coexisted in 6.1% of the isolates.

Conclusion: During the study period, the prevalence of CRE considerably rose in the two tertiary care institutions from western Saudi Arabia. In the CRE isolates, *^bla^*OXA-48 was discovered to be the most common gene. We recommend an antimicrobial resistance surveillance system to detect the emergence of resistant genes through use of new rapid diagnostic tests and monitor antimicrobial use in order to improve clinical outcomes of CRE infections given the severity of infection associated with the CRE isolates as well as the limited treatment options available.

## Introduction

Antimicrobial resistance (AMR) presents a serious global public threat as antibiotics have become increasingly ineffective against drug-resistant organisms. Infections caused by drug-resistant organisms are difficult to treat [1]. The increase in AMR is also associated with limited treatment options, increased morbidity, mortality, and hospitalization duration. Carbapenem-resistant gram-negative bacteria have been flagged on the World Health Organization (WHO) priority pathogen list and there is an urgent need for novel antibiotics to combat such infections [2].

Enterobacteriaceae that are resistant to at least one antibiotic of the carbapenem family or produce carbapenemase are known as carbapenem-resistant Enterobacteriaceae (CRE) [3]. In 2001, Klebsiella pneumoniae carbapenemase (KPC) was initially identified in the United States and is the most prevalent CRE. In addition to KPC, there are several other carbapenemases linked to mobile genetic elements, including Verona integron-encoded metallo-beta-lactamase (blaVIM), New Delhi metallo-beta-lactamase (blaNDM), imipenemase (blaIMP), and oxacillinase-48 (blaOXA-48) [4-8]. In the Gulf Cooperation Council (GCC) countries, specifically Saudi Arabia, the most common carbapenemases are reported to be blaOXA-48, followed by blaNDM [9-11]. A recent study by Hala et al. has identified the first KPC-2-producing CRE in the Gulf region [12].

Currently, carbapenems such as imipenem, meropenem, and ertapenem are among the most potent classes of drugs. In various cases, these drugs are used as the final class of antimicrobial agents to combat infections caused due to multi-resistant Enterobacteriaceae including K. pneumoniae and Escherichia coli (E. coli) [3-8,10,12-15]. The choice of antimicrobial agents used in cases of highly resistant organisms, such as CRE, is limited. Hence, a thorough analysis of the risks and benefits of the available drugs is required. The blaNDM-producing pathogens frequently carry various resistance enzymes like extended-spectrum β-lactamase (ESBL) and AmpC β-lactamases, rendering most antibiotics inactive. However, these organisms are usually susceptible to tigecycline and colistin. Tigecycline has been associated with an increased mortality rate in patients with severe infections and nephrotoxicity [16]. The rise of carbapenem resistance in Enterobacteriaceae has created a generation of organisms that are resistant to nearly all available treatment options [17]. The aim of the study is to estimate the prevalence rate of CRE and to determine the types of carbapenemase genes present in patients admitted to King Abdulaziz Medical City (KAMC-J) and King Abdulaziz University Hospital (KAUH), both in Jeddah, Saudi Arabia.

This article was previously posted to the Research Square preprint server on 26 Aug 2020; however, the authors have requested that this preprint be removed from Research Square.

## Materials and methods

Study design

This retrospective study was performed at KAMC-J and KAUH, Jeddah, Saudi Arabia between 1st April 2017 and 30th March 2019. The Cerner software program (Oracle Cerner Millennium®, Kansas City, MO, USA) was used to extract data on isolated CRE cases, and the data sets were further analyzed using the GeneXpert machine (Cepheid, Sunnyvale, CA, USA).

This study was conducted in compliance with the 18th World Health Congress recommendations (Helsinki, 1964), all its applicable amendments, and the applicable laws, regulations, and guidelines of the Kingdom of Saudi Arabia. The study was approved by institutional review board of the King Abdullah International Medical Research Centre, Ministry of National Guard Health Affairs.

Population

The study data were retrospectively collected from old samples (blood, sputum, urine, swab, wound, fluid, tissue, discharge, nephrostomy, pus, respiratory) of 187 patients who were admitted to either KAUH or KAMC-J. Bacteria belonging to gram-negative Enterobacteriaceae that were phenotypically carbapenem-resistant were included in the study. The demographic information of the patients included in the study were collected using a data sheet.

Inclusion criteria

The samples which were included in the study analysis were isolates of patients ≥ 18 years of age. The samples which developed positive culture for CRE isolates and required further testing by infectious diseases and microbiology professionals considering the clinical deterioration of patients and limited treatment options were included. The samples with CRE isolates exhibited a minimal inhibitory concentration (MIC) of ≥2 mg/L for imipenem or meropenem, based on the Clinical and Laboratory Standards Institute (CLSI) were also included in the study [18].

Exclusion criteria

Samples obtained from pediatric patients were not included in the study. Similarly, repeated isolates from the same patient at the same site and samples with carbapenem-producing organisms other than Enterobacteriaceae were also excluded.

Microbiological testing

Microbiological and molecular testing of the isolated samples were performed at the microbiology department of KAMC-J and the Clinical and Molecular Microbiology Laboratory at KAUH, Jeddah.

Clinical samples (blood, respiratory, swab, urine) of suspected patients were analyzed for gram-negative Enterobacteriaceae using the Vitek 2 system (bioMerieux, Marcy-l'Étoile, France). The breakpoints of imipenem and meropenem were also calculated based on the CLSI guidelines. The selected strains that were identified to be carbapenem-resistant by the Vitek 2 system were further tested for imipenem and meropenem susceptibility by E-test strips (bioMérieux).

Molecular testing of the samples was conducted using Cepheid Xpert Carba-R Assay. The carbapenemase gene families, including blaIMP, blaKPC, blaNDM, blaOXA-48, and blaVIM were identified using Xpert® Carba-R [19].

Statistical analysis

Descriptive statistics were used to describe the demographic and microbiological data of the study. Categorical variables were presented using counts and percentages, whereas continuous variables were presented using mean and standard deviation when data were normally distributed and using median and interquartile range (IQR) when data were not normally distributed. Normality of the numerical data were determined using Kolmogorov-Smirnov test and the Shapiro-Wilk test. Comparison of two groups was done using the one-way ANOVA test or by the Kruskal Wallis test as a non-parametric alternative. The association of the categorical variables was determined using the Pearson Chi-square test or the Fisher exact test.

Prevalence of CRE was calculated based on the antibiograms reports submitted by the two sites participating in the study, KAMC-J and KAUH. Total counts of K. pneumoniae and E. coli were summed up and the percentages of resistance per hospital were calculated.

All mean and median values and their measures of variability were considered up to two decimal places. All percentages were rounded to one decimal place. The significance level was two-sided, with a type 1 error of 5%. The analysis was done using SPSS Statistics for Windows, version 24.0 (IBM Corp., Armonk, NY, USA).

## Results

In this study, 187 patients were diagnosed with CRE infections of which 180 patient isolates met the inclusion criteria. Three of the seven samples that were eliminated belonged to patients under the age of 18, and the remaining four samples included pathogens other than Enterobacteriaceae.

Patients' demographics and characteristics of clinical isolates

The isolates of 180 patients were selected based on the requirement for further testing, given clinical deterioration and the limited treatment options for these patients. K. pneumoniae was the dominant CRE identified in more than 90% of the clinical isolates. Patients' demographics and the characteristics of clinical isolates are depicted in Table 1, while Table 2 shows the distribution of CRE genes in the clinical isolates that were found in different specimens. 

**Table 1:** Patients’ Demographics and Clinical Isolates’ Characteristics SD: Standard Deviation; K. pneumoniae: Klebsiella pneumoniae; E. coli: Escherichia coli; OXA-48: oxacillinase-48; NDM: New Delhi metallo-β-lactamase

Character	NDM	OXA-48	NDM & OXA-48	Neg	Total	P-value (NDM vs OXA-48)
	Mean (SD)/ N (%)	Mean (SD)/ N (%)	Mean (SD)/ N (%)	Mean (SD)/ N (%)	Mean (SD)/ N (%)	
Age	62.9 (16.9)	65.6 (18.8)	67.4 (20.4)	75 (11.7)	62.8 (18.6)	
Male	13 (11.9%)	85 (78%)	8 (7.3%)	1 (0.9%)	109 (60.6%)	
Female	12 (16.9%)	52 (73.2%)	3 (4.2%)	2 (2.8%)	71 (39.4%)	
Hospital						
King Abdul Aziz University	10 (18.2%)	40 (72.7%)	5 (9.1%)	0 (0.0%)	125 (69.4%)	
King Abdul Aziz Medical City	15 (12.0%)	97 (77.6%)	6 (4.8%)	3 (2.4%)	55 (30.6%)	
Organism						
K. pneumoniae	19 (11.4%)	132 (79.0%)	10 (6.0%)	2 (1.2%)	167 (92.8%)	0.007**
E. coli	5 (41.7%)	5 (41.7%)	1 (8.3%)	1 (8.3%)	12 (6.7%)	
Enterobacter	1 (100.0%)	0 (0.0%)	0 (0.0%)	0 (0.0%)	1 (0.6%)	

**Table 2:** Distribution of Carbapenem-Resistant Enterobacteriaceae (CRE) Genes in Isolates Recovered From Various Specimens OXA-48: Oxacillinase-48; NDM: New Delhi metallo-β-lactamase

Resistant isolates	Blood N (%)	Respiratory N (%)	Sputum N (%)	Swab N (%)	Urine N (%)	Wound N (%)	Other N(%)	All Specimen N (%)
OXA-48	46 (33.6%)	8 (5.8%)	34 (24.8%)	12 (8.8%)	16 (11.7%)	9 (6.6%)	12 (8.8%)	137 (76.1%)
NDM	13 (52.0%)	1 (4.0%)	3 (12.0%)	1 (4.0%)	6 (24.0%)	1 (4.0%)	0 (0.0%)	25 (13.9%)
OXA-48 & NDM	3 (27.3%)	1 (9.1%)	3 (27.3%)	0 (0.0%)	2 (18.2%)	1 (9.1%)	1 (9.1%)	11 (6.1%)
Negative	1 (33.3%)	0 (0.0%)	0 (0.0%)	0 (0.0%)	2 (66.7%)	0 (0.0%)	0 (0.0%)	3 (1.7%)
Missing	0 (0.0%)	2 (50.0%)	0 (0.0%)	0 (0.0%)	0 (0.0%)	2 (50.0%)	0 (0.0%)	4 (2.2%)
Total	63 (35.0%)	12 (6.7%)	40 (22.2%)	13 (7.2%)	26 (14.4%)	13 (7.2%)	13 (7.2%)	180 (100.0%)

Susceptibility to different antimicrobials

The K. pneumonia and E. coli isolates were tested for susceptibility against different antimicrobial agents. The clinical isolates were previously identified to be carbapenem resistant and showed resistance to historically well-known antimicrobial agents against gram-negative bacteria. Numerically, a higher proportion of K. pneumoniae isolates were observed to be resistant to carbapenems, trimethoprim/sulfamethoxazole, piperacillin/tazobactam, and ciprofloxacin. Aminoglycosides were also identified to be ineffective. Comparatively, fewer isolates were found to be resistant to tigecycline and colistin. A detailed resistance rate for all isolates is shown in Table 3. Nevertheless, the CRE genes showed different susceptibility to different antimicrobial agents. The isolates with blaOXA-48 gene showed resistance towards carbapenems, trimethoprim/sulfamethoxazole, piperacillin/tazobactam, ciprofloxacin, and aminoglycosides, whereas fewer isolates were resistant to tigecycline and colistin. Additionally, the blaNDM gene was identified to have a similar resistance pattern to that of the blaOXA-48 gene. Isolates with both blaOXA-48 and blaNDM genes were observed to have more resistant isolates. However, their susceptibility to tigecycline and colistin was about 9.1%. The detailed resistance rates of CRE per gene type have been illustrated in Table 4.

**Table 3:** Carbapenem-Resistant Enterobacteriaceae Resistance to Different Antimicrobial Agents K. pneumoniae: Klebsiella pneumoniae; E. coli: Escherichia coli

Antimicrobial Agents	Resistant Isolates			
	K. pneumoniae	E. Coli	Enterobacter	Total
	N (%)	N (%)	N (%)	N (%)
Imipenem	138 (82.6%)	11 (91.7%)	1 (100.0%)	150 (83.3%)
Meropenem	164 (98.2%)	12 (100.0%)	1 (100.0%)	177 (98.3%)
Trimethoprim/sulfamethoxazole	157 (94.0%)	8 (66.7%)	1 (100.0%)	166 (92.2%)
Tigecycline	36 (21.6%)	1 (8.3%)	0 (0.0%)	37 (20.6%)
Colistin	32 (19.2%)	0 (0.0%)	0 (0.0%)	32 (17.8%)
Piperacillin/Tazobactam	167 (100.0%)	10 (83.3%)	1 (100.0%)	178 (98.9%)
Ciprofloxacin	165 (98.8%)	10 (83.3%)	1 (100.0%)	176 (97.8%)
Gentamycin	92 (55.1%)	3 (25.0%)	1 (100.0%)	96 (53.3%)
Amikacin	122 (73.1%)	5 (41.7%)	1 (100.0%)	128 (71.1%)

**Table 4:** Carbapenem-Resistant Enterobacteriaceae Genes Resistance to Different Antimicrobial Agents OXA-48: oxacillinase-48; NDM: New Delhi metallo-β-lactamase

Antimicrobial Agents	Resistant Isolates		
	^bla^OXA-48	^bla^NDM	^bla^OXA-48 & ^bla^NDM
	N (%)	N (%)	N (%)
Imipenem	110 (80.3%)	25 (100.0%)	11 (100.0%)
Meropenem	136 (99.3%)	25 (100.0%)	11 (100.0%)
Trimethoprim/sulfamethoxazole	130 (94.9%)	19 (76.0%)	10 (90.9%)
Tigecycline	31 (22.6%)	5 (20.0%)	1 (9.1%)
Colistin	27 (19.7%)	3 (12.0%)	1 (9.1%)
Piperacillin/Tazobactam	135 (98.5%)	25 (100.0%)	11 (100.0%)
Ciprofloxacin	133 (97.1%)	25 (100.0%)	11 (100.0%)
Gentamycin	71 (51.8%)	16 (64.0%)	7 (63.6%)
Amikacin	102 (74.5%)	16 (64.0%)	11 (100.0%)

Prevalence of CRE

**Figure 1:**
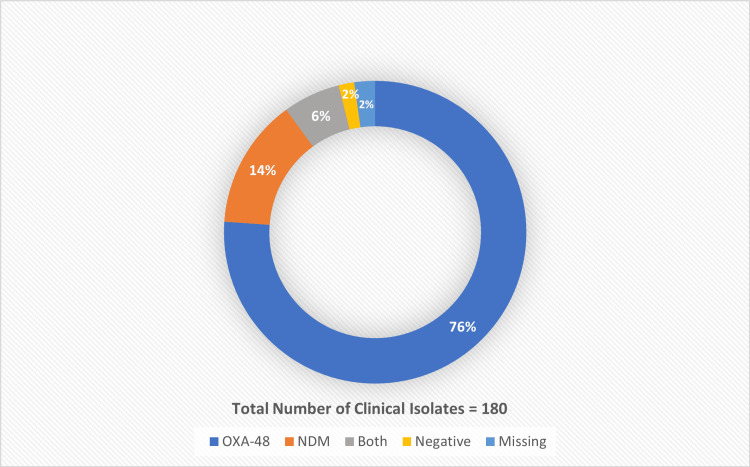
Prevalence of Carbapenem-Resistant Enterobacteriaceae Within Hospital Isolates OXA-48: oxacillinase-48; NDM: New Delhi metallo-β-lactamase

Among the 180 isolates, blaOXA-48 was identified as the dominant resistance gene in 82.2% of isolates, either expressed alone in 76.1% of isolates or coexisting with blaNDM in 6.1% of isolates. No traces of blaKPC, blaVIM, and blaIMP genes were observed among the isolates (Figure 1). K pneumoniae showed a higher frequency of blaOXA-48 genes (79%) versus blaNDM (11.7%) (p = 0.007), whereas E. coli had an equal frequency of both genes (41.7%). 

In this study, presence of the blaOXA-48 gene was identified in samples collected from blood (33.6%), sputum and respiratory tract (30.6%), urine (11.7%), wounds (6.6%), and other specimen sites (8.8%). blaNDM, on the other hand, was identified mainly in the blood (52%), followed by urine (24%), sputum and respiratory (16%), and wound (4%) specimens. The samples containing both genes were obtained from sputum and respiratory (36.4%) specimens, followed by blood (27.3%), urine (18.2%), wounds and others (9.1% each).

**Figure 2:**
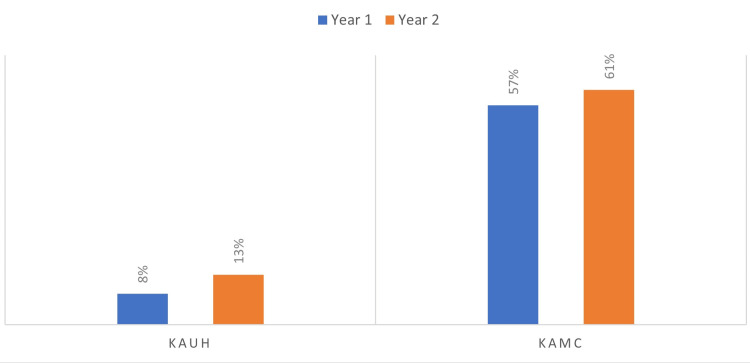
Prevalence of Carbapenem-Resistance Enterobacteriaceae Gene Among Studied Clinical Isolates. KAUH: King AbdulAziz University Hospital; KAMC: King AbdulAziz Medical City. NB: Data for KAMC Year 2 correspond to the period from January 2019 to June 2019 only.

In 2017, 230 out of 2871 Enterobacteriaceae isolates were identified to be carbapenem-resistant at KAUH. In the following year, 339 carbapenem-resistant isolates out of 2602 Enterobacteriaceae were identified by the center, indicating an increase of almost 63% in CRE prevalence. In 2018, 2196 phenotypic CRE were identified out of 3866 Enterobacteriaceae isolates at KAMC. Whereas in the first half of 2019, 1225 phenotypic CRE were identified out of 2008 Enterobacteriaceae isolates, corresponding to a 7.4% increase in CRE prevalence. The trend of CRE resistance of the two hospitals is depicted in Figure 2.

## Discussion

In this study, we reported the difference in the prevelence of CRE infection rates between 2017 and 2019 in western Saudi Arabia. The data indicates a significant increase in CRE infection rate since 2017. blaOXA-48 and blaNDM were found to be the predominant genes responsible for resistance in carbapenemase-producing Enterobacteriaceae (CPE), mainly K. pneumoniae 92.8% and E. coli 6.7%. The most prevalent gene was blaOXA-48, occurring in 82% of isolates either alone or combined with the blaNDM gene. None of the isolates harbored blaKPC, blaVIM, or blaIMP genes. The isolates were found to be resistant to almost all antimicrobial agents except tigecycline and colistin.

The findings of this study complemented the inferences drawn by Alotaibi et al., 2019, which concluded that both blaOXA-48 and blaNDM are the primary resistance-conferring genes produced by CPE, and other genes (blaVIM, blaKPC, or blaIMP) were rarely harbored by CRE. They also reported that K. pneumoniae was identified in 88% and E. coli in 11% of CRE isolates [20]. 

Studies in GCC countries have shown that the most common resistance genes are blaOXA-48 and blaNDM [9,11,21]. This study reported blaOXA-48 (76.1%) to be the predominant resistance-conferring gene followed by blaNDM (13.9%), and blaOXA-48 plus NDM (6.1%), thus concurring with findings of previously published studies. Moreover, a study by Zowawi et al., 2014, also showed similar findings, where 49% of isolates were blaOXA-48-type producers, and 23% were blaNDM-type producers, and six of the isolates coproduced blaNDM and blaOXA-48 genes [11]. Another study from Iran by Solgi et al., 2020, showed the same pattern of predominant organisms and resistance genes [22]. A systematic review on prevalence of antimicrobial resistance in clinical isolates from GCC countries showed that E. coli occurred in 44% of the total isolates, followed by K. pneumoniae in 20%. The study reaffirms the findings that K. pneumoniae and E. coli are the most prevalent pathogens reported from the GCC countries with resistant data [23]. In the current study, K. pneumoniae was reported to be more frequent than E. coli.

Several phenotypic detection methods have been developed to identify and differentiate between the different types of carbapenemases and these methods can be utilized for CRE prevention [9,10]. These methods include growth-based assays, methods of hydrolysis, and lateral flow immunoassays [24]. Studies have shown that blaOXA-48 producers occasionally exhibit low resistance or susceptibility to carbapenems, making the detection of blaOXA-48 producers based on MIC values more challenging [9,25]. For this reason, phenotypic methods can be used to detect carbapenem resistance when the availability of more advanced techniques is absent, whereas molecular methods can be used to confirm ambiguous and negative results [9].

Several studies have reported multi-drug resistant (MDR) CPEs [13,26-27]. Our findings show that K. pneumoniae and E. coli isolates had high resistance to imipenem, meropenem, trimethoprim/sulfamethoxazole, piperacillin/tazobactam, and ciprofloxacin. However, it was also observed that E. coli isolates were more resistant to imipenem, meropenem, and trimethoprim/sulfamethoxazole as compared to K. pneumoniae, while the latter was more resistant to piperacillin/tazobactam, amikacin, tigecycline, and gentamycin than E. coli. Similar findings were observed in a study by Kader et al., 2005, regarding the resistance of E. coli and K. pneumoniae to amikacin; however, resistance to other antimicrobials varied [28]. Although, another study suggested E. coli was to be more resistant to amikacin and gentamycin than K. pneumoniae [29].

CREs, more specifically isolates with blaOXA-48 and blaNDM genes, exhibited a low resistance to colistin and tigecycline as compared to other antibiotics. Although colistin and tigecycline are used as first-line agents against infections caused by CRE pathogens, there are uncertainties regarding their efficacy even when used in combination with other agents [30]. Information regarding sensitivity and resistance of an antimicrobial agent against causative pathogens is crucial as it helps physicians in making clinical decisions. The choice of treatment can also be limited by its affordability. Considering these factors, implementation of antimicrobial stewardship (AMS) is vital to ensure appropriate antibiotic use. Most isolates were found to be resistant against meropenem, trimethoprim/sulfamethoxazole, imipenem, piperacillin/tazobactam, and ciprofloxacin. Nevertheless, the study by Kader et al., 2005, reported that isolates showed the highest resistance with cefepime, gentamicin, and ciprofloxacin [28]. Several studies reported that blaNDM-producers had shown strong resistance to a wide range of antibiotics, including imipenem, meropenem, gentamicin, tobramycin, amikacin, and ciprofloxacin, whereas they were found to be susceptible to colistin and tigecycline [17,31].

Novel antibiotics with new modes of action that could suppress the rise of MDR bacteria are urgently needed. An alternative approach would be to identify molecules that can interfere with the efflux process of the infection-causative organisms. A recent study by Usai et al., 2019 indicated that efflux contributes to microbial strains' overall resistance. The inhibition of efflux pumps by efflux inhibitors (EIs) can enhance the clinical effect of the antibiotics that are their substrates [32].

Although blaOXA-48 pathogens have the ability to mutate rapidly and thereby expand their spectrum of activity [32], less resistance was observed in them [33,34]. These results indicate significant variability in the susceptibility pattern of different CRE isolates to different antimicrobial agents. 

For the patients carrying organisms with two or more CRE genes and second-line treatment options are diminished, indicated by tigecycline/colistin sensitivity (9% versus ~18% with a single gene), future follow-up can be helpful. Genotyping can be used as an important predictor of second-line agents' success or failure.

## Conclusions

During the study period, the prevalence of CRE considerably rose in the two tertiary care institutions from western Saudi Arabia. In the CRE isolates, blaOXA-48 was discovered to be the most common gene. We recommend an antimicrobial resistance surveillance system to detect the emergence of resistant genes through use of a new rapid diagnostic test and monitoring antimicrobial use in order to improve clinical outcomes of CRE infections given the severity of infection associated with the CRE isolates as well as the limited treatment options available.
